# Indocyanine green fluorescence-guided surgery in acute settings: state-of-the art

**DOI:** 10.3389/fsurg.2026.1962138

**Published:** 2026-09-28

**Authors:** Husham Abdelrahman, Mohamed H. Khalaf, Ibrahim Al-Hassani, Mohamed H. Ahmed, Ibrahim Afifi, Fernando Spencer Netto, Ayman El-Menyar, Hassan Al-Thani

**Affiliations:** 1 Department of Surgery, Trauma Surgery, Hamad Medical Corporation, Doha, Qatar; 2 Department of Surgery, Weill Cornell Medical School, Doha, Qatar; 3 Department of Surgery, Qatar University Medical College, Doha, Qatar; 4 Department of Surgery, Recanati/Miller Transplantation Institute, Icahn School of Medicine, Mount Sinai Hospital, NY, United States; 5 Weill Cornell Medical School (Medical Student), Doha, Qatar; 6 Department of Medical Education (Internship), Hamad Medical Corporation, Doha, Qatar; 7 Department of Medicine, Weill Cornell Medical School, Doha, Qatar; 8 Department of Surgery, Trauma and Vascular Surgery, Hamad Medical Corporation, Doha, Qatar

**Keywords:** acute care surgery, anastomotic leak, bowel perfusion, emergency surgery, fluorescence angiography, fluorescence cholangiography, fluorescence-guided surgery, indocyanine green

## Abstract

**Background:**

Operating in acute surgical settings is significantly different from elective surgery. One aspect is the complexity of acutely ill or injured patients with abnormal physiology, unoptimized comorbidities, and unplanned interventions. Traditional visual and tactile assessments of the surgical field anatomy and tissue viability have documented limitations. The need for integrated technology to augment such assessments is described. Indocyanine green fluorescence-guided surgery (ICG-FGS) is a promising tool, given its history, safety, low cost, and accumulated experience in elective settings for many surgeries, to help transform precision surgery into acute settings (trauma and emergency surgery). An updated review is needed based on new evidence outlining the current landscape of ICG in acute settings.

**Methods:**

We performed a comprehensive review of literature update in the last two decades through June 2026, covering PubMed, ClinicalTrials.gov, and published societal guidelines, including, but not limited to, the World Society of Emergency Surgery (WSES) consensus position paper and the Society of American Gastrointestinal and Endoscopic Surgeons (SAGES) review.

**Results:**

ICG is applicable across the full spectrum of acute surgical care, classified under two principal functions: anatomical mapping (biliary, ureteric, thoracic duct, and vascular structures) and perfusion assessment (bowel and mesenteric ischemia, anastomotic perfusion, pancreatic necrosis, burns, soft tissue, extremity trauma, and organ injury). Emerging and less commonly reported applications include necrotizing fasciitis debridement guidance, traumatic brain injury monitoring, penetrating abdominal trauma, gonadal torsion, fistula detection, damage control surgery, and AI-augmented quantitative fluorescence. The WSES consensus and SAGES meta-analysis provided strong prospective validation to date regarding anastomotic leakage reduction. Current gaps include the absence of standardized dosing protocols in acute settings, limited randomized data, and the subjectivity inherent to qualitative fluorescence interpretation.

**Conclusions:**

ICG fluorescence-guided surgery is a safe, low-cost, and rapidly deployable adjunct for emergency surgery. Its breadth of applicability warrants wider consideration and integration into acute surgical workflows to inform surgical decision making based specific considerations including specific indications, evidence support, equipment availability, local expertise, workflow readiness and clinical judgement. Standardized protocols, prospective trials, objective and AI-powered quantification are the research priorities to further clarify its practical role.

## 1 Introduction

Operating in acute settings is significantly different from elective surgery. One aspect is the complexity of acutely ill or injured patients with abnormal physiology, unoptimized comorbidities, unplanned interventions, compressed decision-making time, and challenging operative fields. Anatomy is often distorted by inflammation, bleeding, hematoma, gross contamination, adhesions from previous surgery, and high-stakes uncertainty, creating a risk of misinterpreting critical anatomical structures or assessing vascularity and viability with insufficient confidence (1–3).

Conventional white-light assessment relies on visual and tactile feedback and on the surgeon's experience to interpret anatomy and real-time tissue perfusion. This approach is subjective. Limitations such as obscured tissue planes, edema, inflammation, and poor transferability to trainees support the need for a real-time, objective adjunct that can improve sensitivity, specificity, and reproducibility (1–3, 25).

Indocyanine Green (ICG) fluorescence imaging is a practical tool for intra-operative decision-making. It can complement surgical judgment through two major functions: anatomical mapping and perfusion viability assessment. In acute settings, this may help tailor resections and reconstruction, avoid unnecessary diverting stomas or high-risk anastomoses, and reduce delayed decision-making, missed evolving ischemia, avoidable multiple operations, or reoperation (1–4, 21, 25).

Nevertheless, data from acute care surgery remain less mature than those from elective practice, particularly in trauma and other high-risk settings. Contemporary evidence suggests that ICG fluorescence imaging is feasible, practical, and increasingly relevant in trauma, emergency, and acute care surgery, with the strongest support for fluorescent cholangiography and perfusion-guided intra-operative decision-making (1–3). Nonetheless, the evidence base remains heterogeneous, marked by variations in study designs, clinical indications, implementation protocols, imaging platforms, ICG dose and administration timing, and outcome reporting, as well as limited standardization in patient selection, image interpretation, objectivity, and integration into emergency workflows (2, 3). Consequently, ICG fluorescence imaging should not be viewed as a substitute for surgical judgment but as an adjunct that may strengthen intra-operative assessment when conventional visual and tactile cues are limited or insufficient.

We conducted this updated review to synthesize available evidence, define the current clinical scope of utility across all acute surgical settings, examine operative applications and technical considerations, identify persistent limitations, understand the application framework, and highlight priorities for future investigations in acute settings.

## 2 Methods

We performed a comprehensive literature review in the last two decades through June 2026, covering PubMed, ClinicalTrials.gov, and published societal guidelines, including, but not limited to, the World Society of Emergency Surgery (WSES) consensus position paper and the Society of American Gastrointestinal and Endoscopic Surgeons (SAGES) review.

“This state-of-the-art narrative” review was informed by a structured literature update. We searched PubMed from January 1, 2005, through June 30, 2026, and searched ClinicalTrials.gov and relevant societal guidance up to the same date. Search concepts combined “indocyanine green,” “ICG,” “fluorescence angiography,” “near-infrared fluorescence,” “fluorescence-guided surgery,” “emergency surgery,” “acute care surgery,” “trauma,” “bowel ischemia,” “anastomosis,” “cholecystitis,” “burns,” “necrotizing soft tissue infection,” and related indication-specific terms. Eligible reports described clinical use, implementation, outcomes, technical considerations, or ongoing prospective evaluation of ICG in emergency, trauma, or other acute surgical settings. Elective studies were included only when they provided directly relevant technical or clinical evidence for an acute-care application and were identified as indirect evidence. English-language full-text human studies, systematic reviews, consensus statements, guidelines, and registered clinical trials were considered. Two authors independently screened potentially relevant reports and extracted the indication, setting, study design, sample size, intervention details, outcomes, and limitations; disagreements were resolved by discussion.

Evidence was synthesized narratively because of substantial clinical and methodological heterogeneity. “No formal risk-of-bias tool or certainty-of-evidence framework was applied.”

PubMed was selected as the principal bibliographic database because the review focused on clinically indexed surgical literature, while ClinicalTrials.gov was searched to identify ongoing or recently completed prospective studies and relevant professional-society documents were examined for consensus recommendations. To supplement database searching, we performed backward reference-list screening and forward citation searching of key reviews, consensus papers, and pivotal studies. Nevertheless, reliance on one principal bibliographic database may have excluded relevant non-indexed, non-English, or specialty-database reports. “No formal risk-of-bias tool or certainty-of-evidence framework was applied.”] No data compilation or statistical analysis were performed, and data was synthetized only in qualitative fashion.

## 3 Discussion

### 3.1 Pharmacology, technology, and administration of ICG

Fluorescence occurs when a dye (Fluorochrome) absorbs high-energy light and emits longer-wavelength light. Indocyanine green (ICG) is a water-soluble fluorescent dye developed in 1955 and FDA-approved in 1959. Used clinically for over 60 years, it was first applied to cardiac output measurement, liver function testing, and ophthalmic angiography, and has since expanded into fluorescence-guided surgery (FGS), particularly in hepatobiliary, colorectal, urological, and oncological procedures (25).

ICG is a molecule that rapidly binds to plasma proteins, principally albumin and lipoproteins, upon intravenous injection, restricting it to the vascular compartment. Its half-life in circulation is 3–4 min, after which it is cleared exclusively by the liver via hepatic uptake and biliary excretion. This rapid clearance enables repeated bolus injections within a single operation without signal carryover. ICG sodium salt is available as a powder (typically reconstituted in sterile water to a concentration of 2.5 mg/mL); the reconstituted solution is partially stable and should be used within 6 h of dilution (25).

ICG penetrates tissues by approximately 5–10 mm, enabling real-time imaging of subsurface structures within that range, including bile ducts, vessels, and lymphatics, that are not visible under white light. Prior dissection may help extend this limit slightly for deeper tissues (3, 25).

#### 3.1.1 Near-infraRed (NIR) imaging devices

Near-infrared fluorescence imaging cameras can be built into laparoscopic, open-field, or robotic systems, making them suitable for both open and minimally invasive surgery. Most modern laparoscopes allow surgeons to switch easily between white light and NIR imaging without losing the operative view. Handheld devices also allow ICG use in open surgery, burn units, and emergency departments. Newer systems provide color overlays that combine NIR and white-light images, helping surgeons interpret complex anatomy more easily. The 2025 SAGES guidelines describe the desired features of Fluorescence guided Surgery (FGS) imaging systems (4).

#### 3.1.2 Routes and dosing of ICG

ICG can be delivered intravenously (IV) and through alternative routes, as outlined below. Intravenous injection is used for perfusion assessment and vascular/biliary mapping; the most widely used dose range is 0.1–0.5 mg/kg (typically 2.5–7.5 mg total) administered at least 2–3 min before perfusion assessment or 30 min before biliary imaging. In emergency settings where preparation time is limited, direct intra-biliary (IB) injection into the gallbladder (∼5 mL at 2.5 mg/mL) achieves rapid biliary visualization without the background hepatic signal produced by IV administration. Intra-ureteric injection (2.5 mg/mL, 2 mL per ureter) requires initial cystoscopy and retrograde catheterization. Subcutaneous inguinal injection (0.5 mg/kg) is used for thoracic duct identification (3, 25, 27, 34, 64, 65).

The dosing and timing vary considerably across published series and must be adapted to the clinical context, intended application, imaging device sensitivity, and patient liver function (3). In patients with hepatic impairment, ICG clearance is reduced, background fluorescence increases, and contrast-to-noise ratios for biliary imaging fall. Obesity and significant inflammation similarly degrade image quality. These factors are particularly relevant in the emergency setting, where the patient's hepatic function may already be compromised (3). Figure 1 demonstrates the ICG preparation, indications and interpretations.

**Figure 1:**
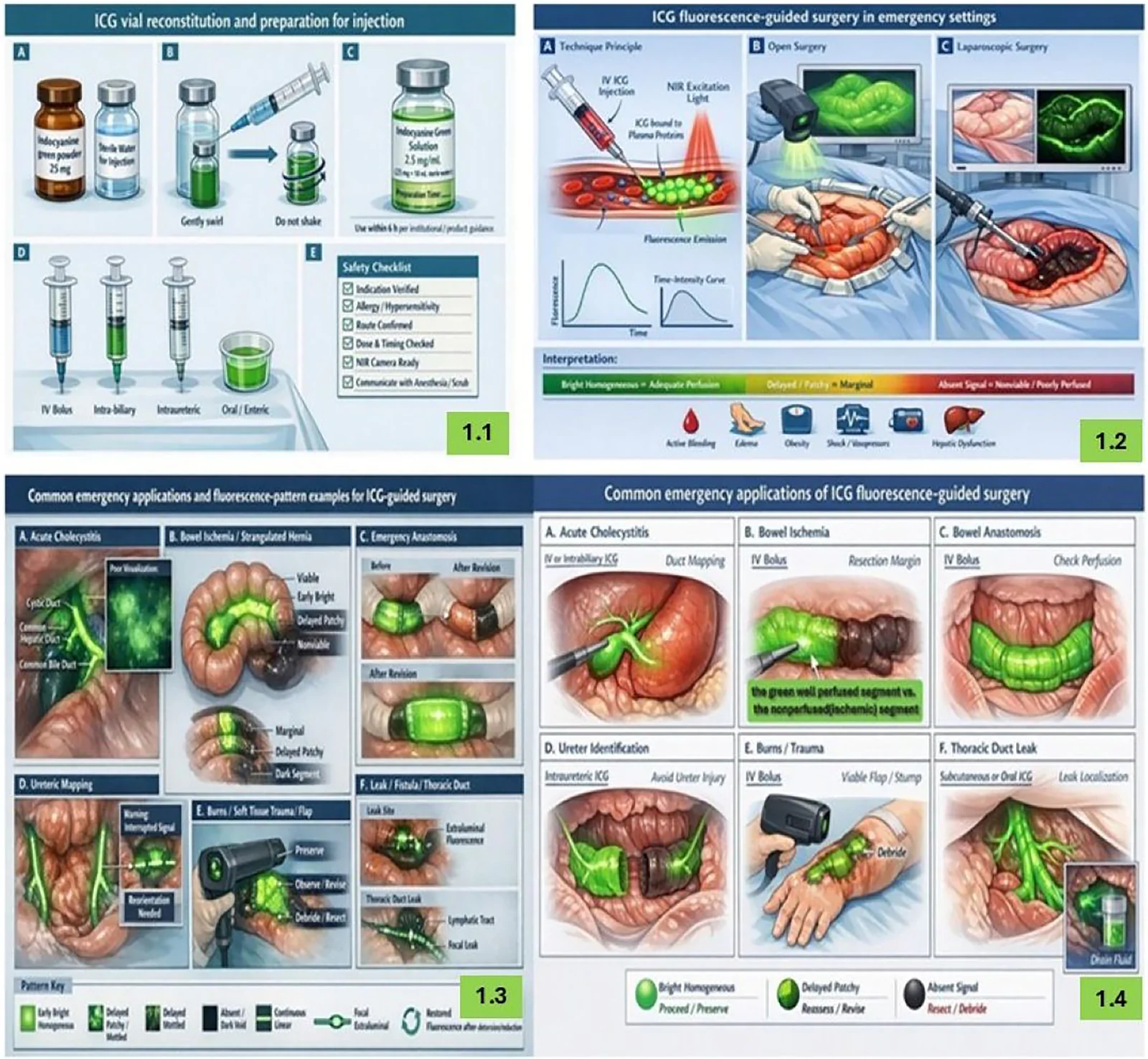
Preparation, principles, interpretation, and acute surgical applications of indocyanine green (ICG) fluorescence-guided surgery: **(1.1):** ICG vial reconstitution and preparation: A: vials, B: gentle mixing, C: reconstituted vial ready to inject, D: different administration routes, E: safety checklist. (**1.2):** Fluorescence-guided surgery principles: A: Technique principle (administration routes, dosing-timing curve), B: Open surgery, C: Laparoscopic surgery. (**1.3):** Interpretation of fluorescence emission and signal patterns: A: Acute cholecystitis, B: Bowel ischemia/strangulation, C: Emergency Anastomosis, D: Ureteric mapping, E: Burn/soft tissue trauma/ Flap, F: Leak/fistula/ thoracic duct. (**1.4):** Representative common emergency applications, including: A: fluorescent cholangiography, B: bowel ischemia, C: anastomotic perfusion, D: ureter identification, E: burn assessment, and F: thoracic duct leak localization.

#### 3.1.3 Safety profile of ICG

ICG has an exceptional safety record across more than 60 years of clinical use. Adverse reactions occur in approximately 1:42,000 administrations and range from mild (urticaria, nausea, hypotension) to rare severe anaphylaxis. The contraindication include allergy to iodine or iodine-based contrast agents,[despite ICG containing trace amounts of iodine (<0.1 mg/mL)],in addition to hypersensitivity to ICG itself. Caution should be taken when planning to administer during pregnancy carries a risk due to potential fetal exposure; however, due to lack of clinical data, it is deemed a relative contraindication. ICG is non-ionizing, has no nephrotoxicity, and is not absorbed by the intestinal mucosa, making it particularly advantageous over radiological contrast agents in the emergency setting. ICG has a very low adverse-event profile, which means the risk-benefit calculation strongly favors its use in selected high-risk emergency procedures (3).

### 3.2 Anatomical mapping in acute surgery settings

#### 3.2.1 Biliary anatomy—acute cholecystitis and hepatic trauma

##### 3.2.1.1 Fluorescent cholangiography (FC) in acute cholecystitis

Bile duct injury (BDI) is the most feared complication of laparoscopic cholecystectomy (LC), occurring in 0.08%–1.5% of cases and carrying significant long-term morbidity and mortality. Acute cholecystitis creates an ideal storm for BDI: a distended, inflamed gallbladder adherent to the common bile duct, obscuring the critical view of safety (CVS). FC with ICG has emerged as a practical alternative to the conventional intra-operative cholangiography (IOC). Unlike IOC, FC does not require cystic duct cannulation, interrupt the operative field, or involve radiation exposure (3, 25–30).

ICG is exclusively excreted into the bile, and IV administration of ICG on induction of anesthesia allows NIR cameras to visualize the extrahepatic biliary tree (including the common hepatic duct, common bile duct, and cystic duct) before and during dissection. Multiple series have demonstrated FC detection rates of 80%–100% for the cystic duct before division. In the acute cholecystitis population, FC has been shown to reduce operative time, lower conversion rates to open surgery, and be associated with a reduction in the incidence of BDI compared with standard white-light LC (3, 26–30). Figure 2 shows the utility of ICG during LC procedure.

**Figure 2:**
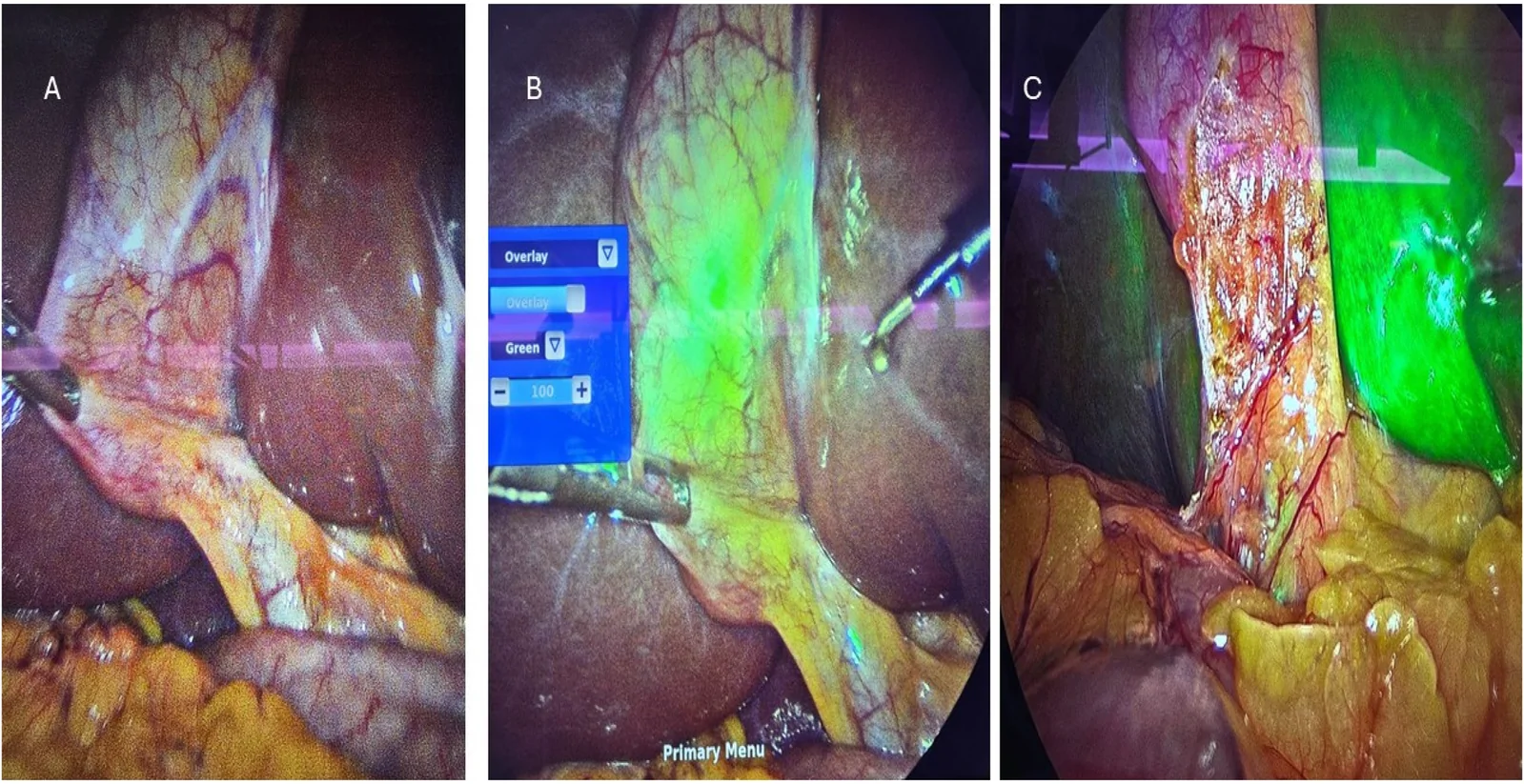
**(A)** Lap view of the inflamed gall bladder prior to dissection under white light. **(B)** ICG-cholangiogram demonstrating the common bile duct fluorescence which can guide and streamline safer dissection of calots triangle. **(C)** ICG cholangiogram during the dissection denoting the CBD.

Although the IV route is standard, severe inflammation can occasionally cause high hepatic background activity, leading to inadequate imaging. In these technically challenging cases, direct intra-biliary injection into the gallbladder (5 mL of a 2.5 mg/mL solution) improves the target-to-background ratio and allows visualization of the biliary tree; however, it may be ineffective if an impacted stone blocks the cystic duct (3, 27).

Beyond standard cholecystectomy, ICG can also be a beneficial adjunct in patients requiring laparoscopic transcystic common bile duct exploration, shortening operative times and leading to faster recovery. Furthermore, the use of ICG can shorten the learning curve for general surgery trainees performing cholecystectomy (28, 30).

IV administration for the induction of anesthesia is an acceptable timing for the IV injection before starting cholecystectomy, with reports showing no difference in achieving visualization of the hepatocystic triangle with early or delayed administration. Evidence from randomized and comparative studies suggests that the optimal view is achieved when the dye is injected 2–3 h before the procedure, allowing adequate excretion of the dye in the bile. If feasible, early administration is favorable; however, it should not deter surgeons from using ICG when administered on the induction (which generally allows for 30 min passage before acquiring the imaging) or intra-biliary during the procedure. Reports showed no difference in achieving visualization of the hepatocystic triangle with administration 2-3 h hours before the procedure (26, 27, 29).

The 2025 WSES consensus provides its strongest recommendation (Grade 1C) for the use of ICG FC in acute cholecystitis, noting that it facilitates early identification of relevant extrahepatic biliary anatomy, achievement of the Critical View of Safety (CVS), reduces operative time, and lowers biliary duct injury rates, though it based on low level evidence. The same consensus provided a step-by-step protocol for effective ICG FC in the emergency setting, stipulating laparoscope positioning at 90 degrees to Calot's triangle, distance < 5 cm from target structures, and the preference for direct IB injection in severely inflamed cases (3).

Recent randomized and cohort evidence has further refined the role, route, and timing of ICG FC in laparoscopic cholecystectomy, including comparative route studies, DOTIG (Dose and administration Time of Indocyanine Green in near infrared FC during LC) timing data, and fixed or weight-based dosage seems to be both effective and learning-curve evidence (26–30). Nevertheless, evidence with this application of ICG FC is based on predominantly elective evidence, while direct acute care is based on single-center experiences and case series.

##### 3.2.1.2 Biliary leak detection after damage control surgery for hepatic trauma

Severe hepatic trauma managed by damage control surgery (DCS) creates a high-risk population for occult biliary leaks during the index or second-look laparotomy. ICG fluorescent cholangiography—administered intravenously 30 min before surgery—can help identify transected intrahepatic and extrahepatic ducts that may not be apparent on white-light inspection or in a contaminated operative field. This technique has been reported in elective hepatic biliary and live liver donation surgery; thus, it is predominantly elective evidence. It is theoretically applicable and potentially highly valuable in the trauma setting, where a missed biliary leak mandates reoperation. Consensus groups have noted this as an area warranting prospective study, noting that timing constraints in trauma may necessitate adaptation of dosing protocols (3, 31, 32).

#### 3.2.2 Ureteric identification

Ureteric injury is a serious complication of emergency colonic resection for diverticulitis, sigmoid volvulus, and pelvic trauma, with an incidence reported up to 5% in colorectal surgery. Intra-ureteric retrograde injection (2.5 mg/mL, 2 mL per ureter) of ICG allows real-time fluorescence identification of both ureters throughout the operative field using standard NIR-capable laparoscopes. Successful ureteric identification rates are high in reported series and systematic reviews, with visualization commonly achieved intra-operatively, and some reports demonstrate persistent ureteric fluorescence for up to eight hours. Although intra-ureteric ICG may still require cystoscopy with or without ureteric catheterization, injection-only approaches may reduce stent-related time and morbidity, which is particularly relevant in emergency settings where stent placement adds time and requires urological assistance (64, 65). Evidence is predominantly elective.

More recent work has explored non-invasive ureteric-specific fluorophores that are excreted renally, permitting ureteric visualization without catheterization; a potential major advantage in emergency environments. Although not yet in clinical practice, this represents a near-term direction for noninvasive ureteric mapping (33, 65).

#### 3.2.3 Thoracic duct mapping—traumatic chylothorax

Thoracic duct (TD) injury results in chylothorax, a rare but morbid complication of thoracic trauma, esophageal surgery, and central venous line placement. Intra-operative identification of the site of TD leakage using conventional white-light thoracoscopy is challenging. Subcutaneous injection of ICG (0.5 mg/kg) 30–60 min before thoracoscopy opacifies the thoracic duct via lymphatic uptake, enabling the surgeon to identify the leakage site and achieve definitive ligation. Vecchiato et al. reported this technique in 19 patients undergoing minimally invasive esophagectomy, with no adverse effects and reliable TD identification at a mean of 52.7 min from injection. Extrapolation to traumatic chylothorax managed via video-assisted thoracoscopy (VATS) is logical and represents a promising acute care application not previously reported in the trauma literature (34).

#### 3.2.4 Perfusion assessment in acute settings

##### 3.2.4.1 Bowel and mesenteric ischemia

###### 3.2.4.1.1 Acute mesenteric ischemia

Acute mesenteric ischemia (AMI), whether occlusive (arterial embolism or arterial or venous thrombosis) or non-occlusive (NOMI), is among the most devastating abdominal emergencies, with mortality exceeding 50% in many series. Surgical intra-operative demarcation of viable from non-viable bowel directly determines the extent of resection, whether anastomosis is safe, and whether a second-look procedure is required. Visual assessment of bowel color, palpable peristalsis, and mesenteric bleeding has low sensitivity and high inter-observer variability, especially in NOMI, where tissue changes may be subtle and patchy (3, 9, 10).

ICG-FA provides real-time perfusion mapping that directly addresses this limitation. Following intravenous (IV) bolus administration (2.5–7.5 mg), the fluorescence signal appears within 30–60 s, allowing the surgeon to delineate zones of perfusion and hypoperfusion at resection margins, guide the decision to proceed with or defer anastomosis, and confirm adequate marginal perfusion after revascularization. In Karampinis et al.'s series of patients with AMI, ICG-FA provided additional information to macroscopic assessment, demonstrating its additive value over standard assessment. Joosten et al. and subsequent case series confirm that ICG-FA can change operative management, extending resection in some cases and preserving bowel in others (3, 9, 10).

The 2025 WSES consensus makes a strong recommendation (1C) for intra-operative decision-making in patients presenting with both occlusive and non-occlusive mesenteric ischemia, citing its ability to evaluate intestinal blood flow, define resection margins, and assess anastomotic safety, though it is based on low-level evidence (Case series and retrospective studies). The prospective FLAMES trial (NCT06322862), a multicenter cohort study of ICG-FA in all-cause bowel ischemia presenting as a surgical emergency, is currently recruiting and will provide the first structured prospective data on this application (3, 70). Figures 3–5 demonstrate the utility of ICG in acute bowel surgeries.

**Figure 3:**
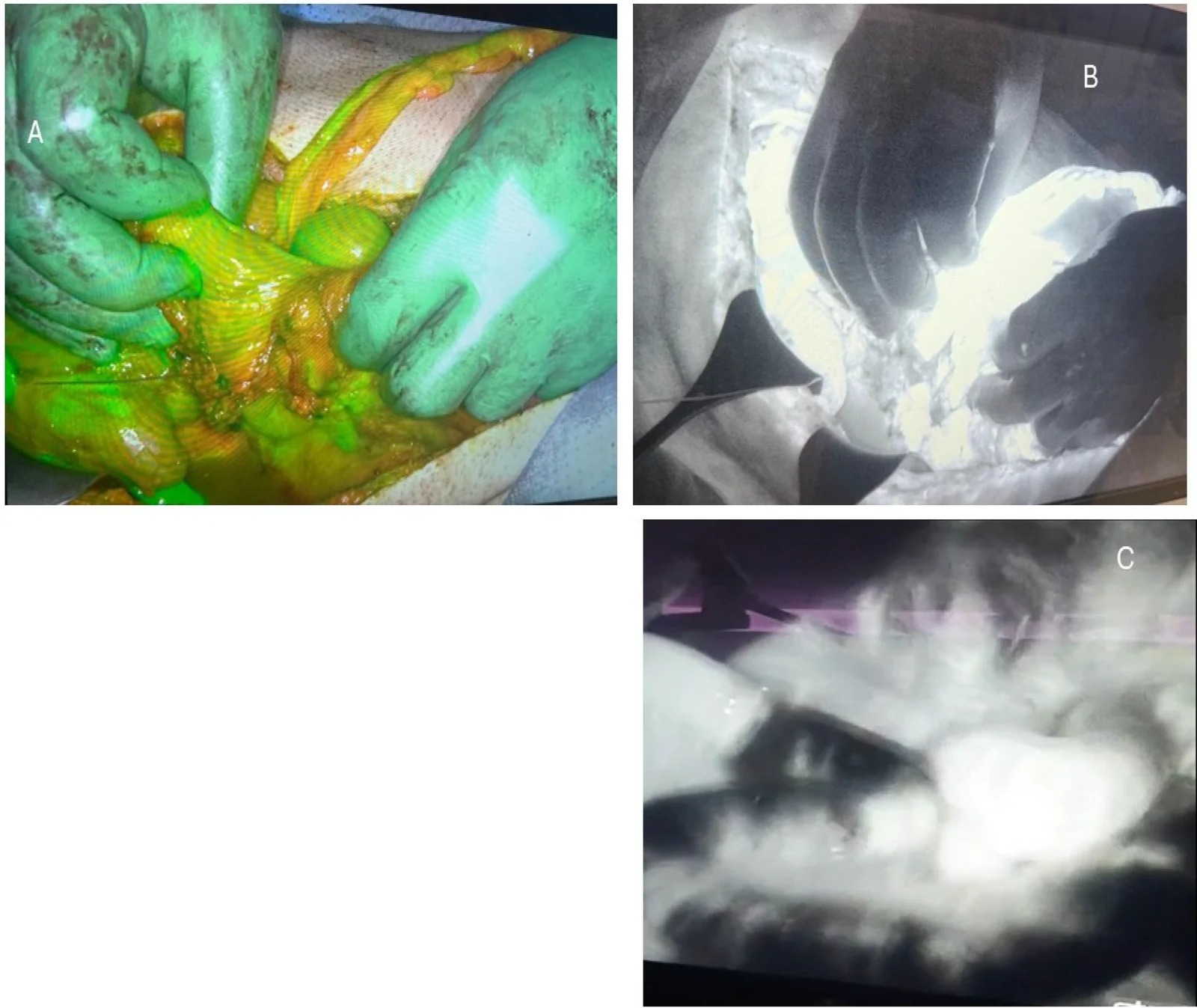
**(A)** Fluorescence-guided assessment of anastomosis post reconstruction to document perfusion **(B)** Fluorescence-guided assessment of anastomosis post reconstruction to document perfusion. ICG fluorescence image showing perfusion heterogeneity within the operative field. **(C)** The contrast between stronger and weaker fluorescence helps identify potentially viable and threatened bowel regions (segment) during acute mesenteric ischemia.

**Figure 4:**
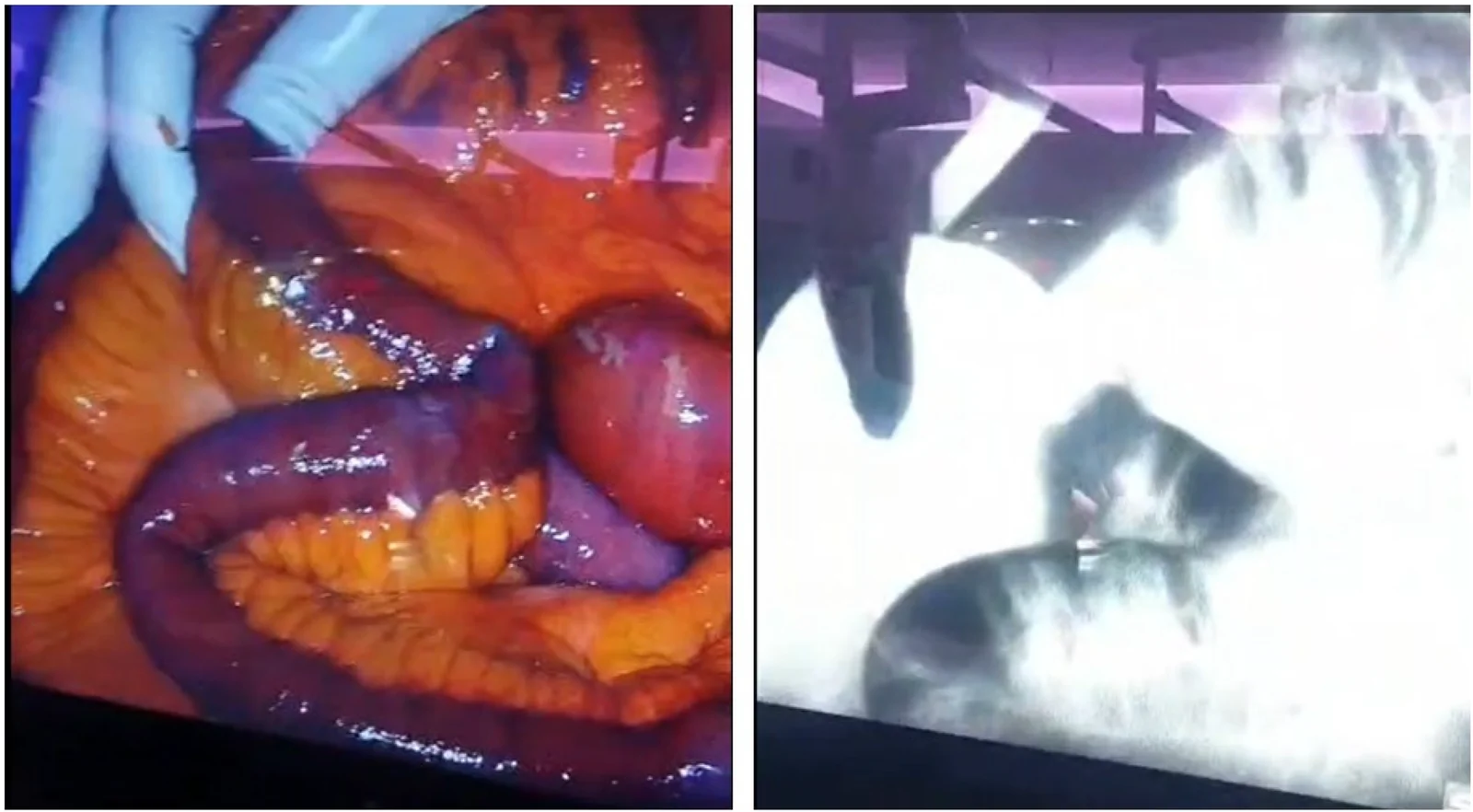
ICG perfusion assessment: notice the contrast between well-perfused bright bowel and the ischemic segment of bowel to be resected. Real-time white light assessment of same segment with observed fixed color changes and congestion early gangrene.

**Figure 5:**
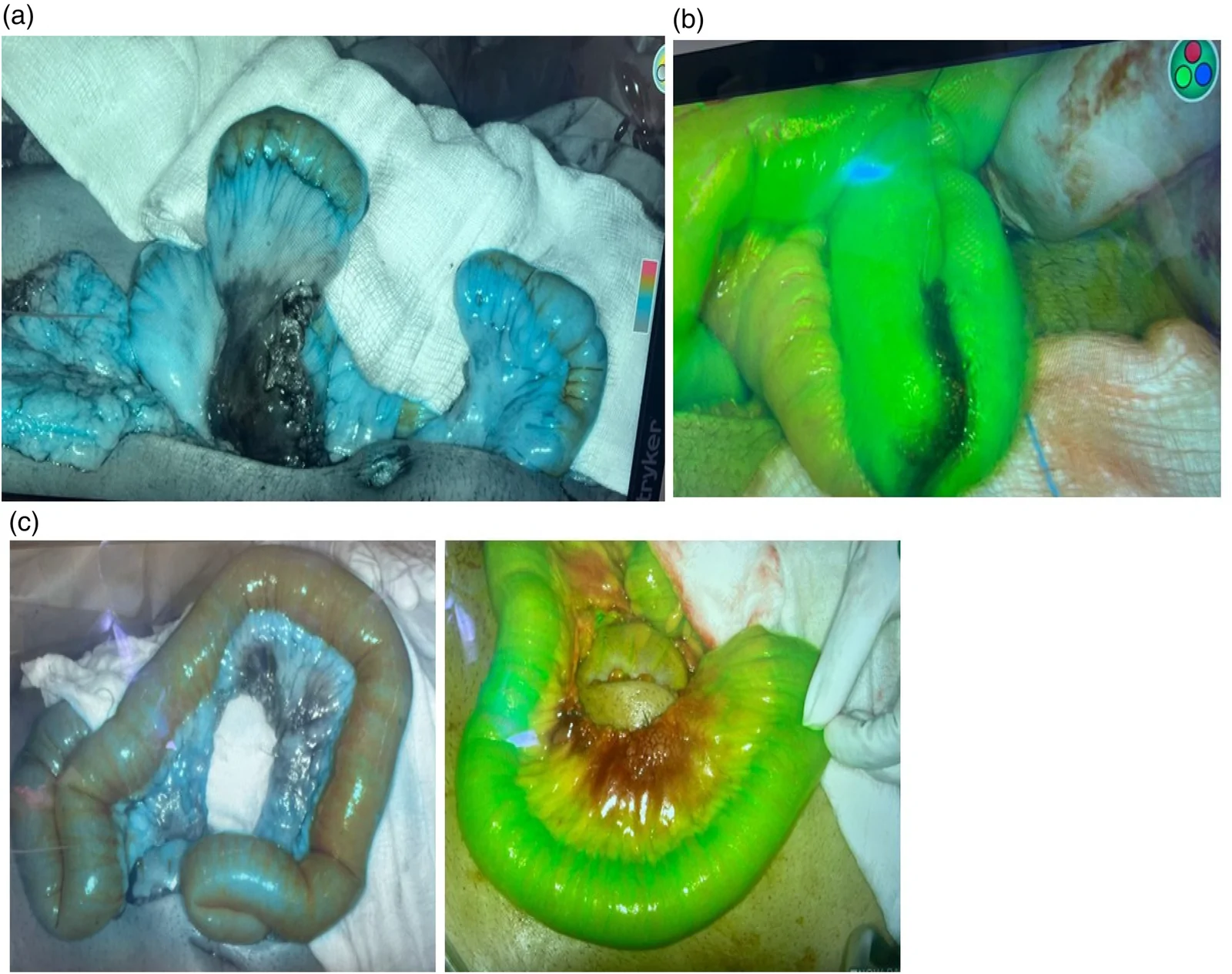
**(a)** Representative near-infrared fluorescence image showing ICG-guided assessment in an acute operative field. The fluorescent signal identifies the target area (post-resection bowel ends; note the mesenteric hematoma post-traumatic) and supports intra-operative interpretation. **(b)** Fluorescence-assisted visualization of anastomosis to document perfusion real-time. **(c)** ICG fluorescence assessment of bowel or visceral perfusion at a critical decision point (Non-devascularizing mesenteric traumatic tear). Stronger fluorescence indicates preserved vascular supply (no need to resect). Same patient using the green mode.

###### 3.2.4.1.2 Strangulated bowel and incarcerated hernia

Bowel strangulation in the context of an incarcerated hernia, internal hernia, closed-loop obstruction, or volvulus presents the same perfusion assessment dilemma as AMI, with the added complexity that releasing the strangulation may lead to partial or complete reperfusion, making immediate resection decisions unreliable. Several series have documented cases in which bowel that appeared grossly ischemic by conventional criteria demonstrated adequate ICG perfusion after hernia reduction, allowing avoidance of unnecessary resection. Conversely, apparently viable bowel may be critically hypoperfused by ICG, prompting extended resection to reduce anastomotic failure (10).

Recent evidence of emergency bowel ischemia supports this role. Christofi et al. reviewed 11 studies on emergency bowel ischemia involving 358 patients. At the same time, Nicolae et al. reported that ICG-FA changed surgical strategy in 29%–67% of acute bowel ischemia cases across the included studies. Noguchi et al. described two cases of strangulated small bowel obstruction in which ICG fluorescence imaging guided intestinal preservation and perfusion assessment during laparoscopic emergency surgery (67–69). Thus further research is needed to build stronger evidence and define the three potential roles (first precision in recognizing need for resection and the extent, second expand application of minimally invasive approaches in these cases without the need to convert to open, and third inform intra-operative decision making for less trained) in addition the improve rigorous objectivity of the interpretation of the imaging to minimize misinterpretations (3).

###### 3.2.4.1.3 Cecal volvulus and sigmoid volvulus

ICG-FA has been reported in the emergency management of cecal volvulus, where it confirmed bowel viability after reduction and guided the decision between cecopexy and formal resection. When ischemia is present, ICG delineates the extent of nonviable tissue with greater precision than white-light inspection. This is particularly relevant in sigmoid volvulus, where the extent of viable sigmoid colon remaining after detorsion determines the feasibility of primary anastomosis vs. Hartmann's procedure (10, 21).

#### 3.2.5 Anastomotic perfusion and leak reduction

Anastomotic leakage remains a leading source of surgical morbidity and mortality following colorectal resection, with rates ranging from 2.8% to 19% depending on the anatomical location. Hypoperfusion at the resection margins is a primary modifiable risk factor. ICG-FA enables direct real-time assessment of perfusion at the planned anastomotic site before and after anastomosis construction, allowing surgeons to modify the resection level if perfusion is inadequate (4, 5, 35).

The evidence base for this application has matured substantially since prior original reviews. The SAGES 2025 systematic review and meta-analysis included seven RCTs and demonstrated that ICG-FA significantly reduced left-sided colorectal anastomotic leak rates. A separate randomized-trial meta-analysis also confirmed significant leak reduction, with the greatest benefit observed in left-sided and rectal resections. Notably, the ICG-COLORAL RCT (Rinne et al., JAMA Surgery 2025; *n* = 1,136) did not demonstrate a significant reduction in anastomotic leak rate when low anterior resections were excluded, suggesting that the benefit of ICG-FA may be primarily concentrated in left-sided and rectal anastomoses where perfusion is most vulnerable (4, 5, 35).

This application warrants separate consideration from generic bowel perfusion assessment because it has one of the strongest evidence bases for extrapolation from elective colorectal surgery to trauma-related colonic resection and anastomosis. Trauma patients undergoing colonic reconstruction often have higher baseline leak risk due to contamination, shock, transfusion, vasopressor exposure, and abbreviated decision-making. In this context, ICG-FA may provide a practical, real-time adjunct to confirm anastomotic perfusion and justify primary anastomosis or diversion decisions in high-risk bowel trauma. The strong recommendation is based on low evidence to consider it in emergency resections anastomosis by the consensus paper (3, 4, 8, 35)

#### 3.2.6 Pancreatic tissue viability in necrotizing pancreatitis

Surgical management of infected or walled-off pancreatic necrosis (WOPN) requires the accurate delineation of viable and necrotic pancreatic parenchyma to guide the extent of necrotic tissue debridement (necrosectomy). Overaggressive debridement risks hemorrhage, pancreatic fistula, and endocrine insufficiency, whereas under-debridement leaves infected necrotic tissue. Intravenous administration of ICG during video-assisted retroperitoneal debridement (VARD) or transgastric endoscopic necrosectomy allows real-time visualization of viable pancreatic tissue, which fluoresces brightly in contrast to dark, avascular, necrotic areas. Case reports and technical notes have described this application, including Brito-Carmona et al. laparoendoscopic treatment of walled-off pancreatic necrosis which is a direct acute care evidence. ICG is not yet the standard of care for pancreatic necrosectomy, but the physiological basis is sound, and further prospective evaluation is warranted (3, 36).

### 3.3 Skin, soft tissue, and burns

#### 3.3.1 Burn depth assessment

##### 3.3.1.1 Burn injuries

The management of burn wounds, including the size of donor graft, is guided by the depth of dermal tissue involvement (46). However, interpretations of burn depth currently depend on the subjective assessment by a treating physician, which is accurate in only 60%–75% of cases (47). In addition, unnecessary autograft usage can lead to significant donor site morbidity, including scarring, pain, and infection (48). Intra-operative burn depth assessment is therefore a critical factor in reducing unnecessary autograft (46–50). This has provided the clinical rationale for objective perfusion-based tools, such as ICG fluorescence angiography (46, 49, 50).

Multiple studies have provided strong evidence supporting the use of ICG for burn depth assessment. A multicenter prospective cohort study conducted by Wongkietkachorn et al. (49) reported that ICG angiography had a sensitivity and specificity of 100.0% in recognition of burn depth, while standard clinical assessment yielded a sensitivity of 33.3% and a specificity of 66.7%. Additionally, Junak et al. (46) demonstrated that second-window ICG (which is a technique to enhance visualization) intensity correlates with histologic burn depth.

Interestingly, a systematic review of nine studies found that while ICG was accurate in distinguishing viable and non-viable tissues, differentiation between superficial and deep partial-thickness burns was inconsistent in some studies (50). Thus, ICG may be more useful in the context of necrosectomies to preserve viable tissues. However, this systematic review was unable to analyze the data quantitatively due to methodological heterogeneity. Therefore, future prospective studies with standardized ICG protocols are needed to clarify the indications and diagnostic capabilities of ICG in burn depth assessment. ICG angiography for burn depth assessment reduces unnecessary excisions, reduces inadequate excisions, and potentially improves healing outcomes and donor-site morbidities (3).

#### 3.3.2 Amputation level and flap viability

##### 3.3.2.1 Use of ICG in amputation and debridement

Most patients with lower extremity amputation (LEA) undergo multiple rehospitalization events months to years after surgery due to complications such as infection, wound healing issues, and hematomas in 49%–52% of cases (38, 39). Most of these complications are related to low tissue perfusion at the amputation stump, making determination of the optimal amputation level difficult and increasing the risk of eschar formation (40, 41). Therefore, the use of ICG angiography for tissue viability assessment in the context of LEA may prove useful (15, 40–45).

Numerous studies have shown that ICG angiography is effective in predicting post-operative wound healing, flap viability, and tissue necrosis. Yang et al. conducted a retrospective study of 17 amputations and found that intra-operative relative perfusion values below 31% had 100% sensitivity and specificity for predicting necrosis (15). Similarly, a prospective cohort study of 15 amputations showed that four cases with low-fluorescence regions on ICG angiography were associated with skin necrosis in all cases, with three of the four requiring re-amputation. Ten out of the 11 wounds with normal fluorescence healed without complications (41). Zetzmann et al. demonstrated that in stumps requiring revision, ICG angiography showed significantly lower perfusion values (42). The strong predictive capability of ICG angiography may allow surgeons to revise to a more proximal, better-perfused level during the index operation, reducing readmission due to perfusion-related complications. Green et al. demonstrated that intra-operative ICG angiography allowed surgeons to make revisions in 31.7% of amputation stumps (16).

As with amputation, the use of intra-operative ICG angiography during debridement procedures may also reduce perfusion-related complications. A prospective study evaluating laser-assisted ICG angiography in Gustilo IIIB open lower-limb fractures showed significantly lower rates of tissue necrosis and deep-site infections, as well as fewer debridement sessions (43). Likewise, Fang et al. found that in patients with diabetic foot ulcers, ICG angiography identified necrotic tissue with a 96% positive fluorescence rate and was associated with fewer debridement sessions (44).

Overall, the use of ICG in the context of amputation and debridement is promising and may be underutilized, specifically in lower extremity injuries (45). However, the lack of randomized controlled trial data and the absence of standardized perfusion thresholds across devices or software have made implementation challenging.

### 3.4 Extremity trauma, vascular injury, and war-related wounds

Vascular injury to the extremities, caused by blunt trauma, penetrating injury, blast, or iatrogenic causes, presents the surgeon with the task of confirming successful revascularization and predicting intra-operative complications. Intra-operative ICG angiography can be performed after vascular repair to confirm flow restoration and identify residual ischemic zones. It can also be performed in the ICU or ward setting to monitor perfusion noninvasively in patients with vascular reconstruction, those at risk of compartment syndrome, or those under observation for limb salvage (15, 16, 23).

In war-related traumatic injuries, which are characterized by high-energy transfer, heavily contaminated wounds, and blast-associated soft tissue destruction, ICG angiography has been reported by Green et al. in a case series in which 19% of patients required operative modification for better-perfused tissues based on ICG findings. Nine patients used ICG for bowel perfusion in this series. This technique is particularly valuable in a military or mass-casualty context, where rapid, bedside-deployable, non-ionizing perfusion assessment allows triage of borderline limbs and wounds (16).

### 3.5 Emerging and novel applications

#### 3.5.1 Penetrating abdominal trauma

Penetrating hollow visceral injuries (gunshot or stab wounds) have recently been described as a setting in which ICG-FA can inform decisions related to resection extent, anastomosis, or diversion. Unlike blunt trauma, penetrating injury delivers concentrated kinetic energy to the bowel and mesentery, creating zones of microscopic ischemia extending beyond the visible wound margins. These zones of occult perfusion compromise are directly amenable to ICG assessment (6).

Patel et al. published the first comparative case-control study of ICG-FA in penetrating abdominal trauma at a Level 1 Trauma Center in Cape Town, South Africa. In 20 patients who underwent laparotomy with ICG-FA vs. 80 who did not, the overall complication rate was significantly lower in the ICG group. A feasibility trial (NCT04534816) completed in 2024 confirmed that ICG-FA changed operative management in penetrating abdominal trauma cases and was usable and clinically relevant. A parallel case report described diagnostic laparoscopy with ICG fluorescence testing to exclude bowel ischemia in blunt abdominal trauma with mesenteric hematoma, avoiding intestinal resection and temporary ostomy (6).

These results establish ICG-FA as a particularly valuable tool for penetrating abdominal trauma and likely for blunt as well, a population in which the consequences of a missed ischemic margin or an unnecessary stoma are severe (6).

#### 3.5.2 Necrotizing fasciitis and soft tissue infection debridement

##### 3.5.2.1 Use of ICG in necrotizing soft tissue infections (NSTIs)

NSTIs are life-threatening infections with a mortality rate of approximately 30% (51–55). Early diagnosis and intervention are essential and have been shown to reduce mortality (56). However, identifying NSTI based on clinical signs and symptoms is challenging and can be mistaken for cellulitis, as NSTI is often nonspecific (57). The current standard for identifying NSTI is the Laboratory Risk Indicator for Necrotizing Fasciitis (LRINEC) score, with a sensitivity and specificity of 68% and 85%, respectively (58). ICG angiography may prove useful as a clinical tool to improve the accuracy of NSTI identification.

The principle underlying the use of ICG in NSTIs is that the pathophysiology involves microvascular thrombosis caused by bacterial exotoxins (54). This subsequently decreases perfusion and appears as a fluorescence signal void (Dark non fluorescent areas) in any affected tissue. In contrast, cellulitis appears with increased fluorescence (hyperemia) and the absence of signal voids. Galante et al. described the use of ICG fluorescence perfusion assessment as a surgical adjunct in necrotizing fasciitis management (11). Ray et al. (59) supported this theory and demonstrated that all kinetic fluorescence parameters (signal to background ratio(SBR), Maximum fluorescence intensity, and Ingress Slope, except Time To Peak intensity, could distinguish between NSTI and cellulitis. No statistically significant difference was observed in the LRINEC scores between the two groups. The sensitivity and specificity for ICG angiography in the study by Ray et al. (59) were 100% and 75%, respectively. Similarly, Streeter et al. (60) reported signal voids in patients with NSTI.

However, no published clinical trials have evaluated the usefulness of ICG in NSTIs. Therefore, further research is warranted to confirm the viability of ICG in NSTIs before any recommendations can be made. Two ongoing clinical trials, NCT04839302 and NCT06877793, are testing ICG kinetic modeling to diagnose necrotizing soft tissue infections and guide real-time surgical debridement in patients with suspected NSTI (61, 62).

#### 3.5.3 Traumatic brain injury and neurocritical care

ICG near-infrared spectroscopy (NIRS) allows real-time measurement of cerebral perfusion at the bedside without ionizing radiation or the need for surgical access (13, 14, 37).

In surgical evidence, Kamp et al. used ICG cortical perfusion assessment during decompressive craniectomy for severe TBI and correlated fluorescence curve parameters with the Glasgow Outcome Scale and modified Rankin Scale at 3 months (12).

The ICG-derived fluorescence curve differed significantly between patients with favorable and unfavorable outcomes, reflecting the underlying pathophysiology of elevated ICP, capillary leakage, and venous congestion (12).

Non-invasive ICG NIR spectroscopy, which is currently being investigated for subarachnoid hemorrhage (SAH) and ischemic stroke, has the potential for ICU neuromonitoring without requiring surgical access. Forcione et al. reviewed the literature on ICG-enhanced cerebral perfusion assessment in brain trauma. They concluded that, while current devices have limitations, this technology can identify clinically important biomarkers and delineate pathophysiological changes in real time. Further large studies are needed to determine the role of TBI management algorithms (13, 37). Thus, the evidence for monitoring is extrapolated from other nonsurgical emergencies.

#### 3.5.4 Gonadal torsion

Testicular and ovarian torsion are surgical emergencies in which the operative decision (detorsion and organ preservation vs. orchiectomy/oophorectomy) depends on the viability of the gonadal tissue after detorsion. Conventional assessment relies on color change, pulsatile arterial flow on Doppler, and clinical judgment, all of which are highly subjective. ICG angiography (2.5 mg IV) allows direct real-time visualization of gonadal perfusion within approximately 60 s of injection after detorsion, providing an objective basis for preserve-or-resect decision-making (17, 63).

Nicholson et al. reported 12 cases of ovarian torsion where ICG demonstrated return of perfusion in 9 patients, guiding organ-preserving surgery (17). In the context of testicular torsion, Shirasaki et al. reported successful testicular salvage guided by ICG fluorescence (63). These findings are of particular clinical relevance in the pediatric and reproductive-age population, where unnecessary gonadectomy carries long-term consequences (17, 63).

#### 3.5.5 Damage control surgery and second-look laparotomy

The damage control surgery (DCS) paradigm (initial hemorrhage and contamination control with planned second-look laparotomy after resuscitation) creates a specific scenario in which ICG-FA can add value to both the index and re-look operations. At the index DCS, ICG-FA can confirm which bowel segments are critically ischemic (warranting immediate resection vs. temporary closure of the abdomen) and which retain perfusion adequate to survive the resuscitation interval. At second-look laparotomy, ICG-FA provided updated perfusion data after resuscitation, correcting hemodynamic derangements that had distorted perfusion patterns during the index operation (3).

The sequential use of ICG across the DCS stages represents a novel and highly practical application. It has the potential to reduce both unnecessary ostomy creation and the risk of anastomotic failure in a population that is exceptionally at high risk for both (3).

#### 3.5.6 Intestinal fistula post-operative leak confirmation and stoma viability

Because orally administered ICG is not absorbed by the intestinal mucosa, it remains within the bowel lumen and can extravasate through an anastomotic defect or fistulous tract. ICG fluorescence has therefore been reported as a simple, bedside-deployable method for confirming postoperative anastomotic leak or intestinal fistula. Peng et al. described oral ICG administration with near-infrared detection of fluorescence in drain fluid as a real-time fistula test. This noninvasive, radiation-free approach can be performed at the bedside without anesthesia. It may be useful for postoperative monitoring in trauma patients or as a triage tool before surgical re-exploration (20).

An extrapolated use that needs to be studied further is using ICG to assess stoma viability before or after maturation to avoid complications of ostomy necrosis or suture line disruption. ICG has been used to assess ileal conduit viability before ureteric implantation, primarily to assess the ureter; nevertheless, the bowel can be assessed in the same setting. A similar technique can be applied with the ostomy where the mesentery is taut, or the abdominal wall is thick, putting tension on the mesentery. An IV injection of ICG can be used to assess perfusion prior to abdominal wall closure.

#### 3.5.7 Appendiceal base assessment

ICG-FA has been used to assess the perfusion of the appendiceal base before appendectomy and the stump after completion of the appendectomy. Zorzetti et al. reported five cases in which ICG-FA confirmed adequate stump perfusion, guiding the closure technique used. Although routine appendectomy rarely requires perfusion assessment, this application may be relevant in cases of base involvement by inflammation, inadvertent vascular injury, or Crohn's disease, where stump breakdown is a recognized risk factor (22).

#### 3.5.8 Partial splenectomy and splenic trauma

Spleen-preserving surgery, whether for benign splenic lesions or in the management of Grade III–IV splenic lacerations with attempted splenic salvage, requires real-time confirmation that the residual splenic parenchyma is adequately perfused. ICG angiography allows the surgeon to confirm arterial inflow and venous outflow to the splenic remnant after selective vascular clamping, guiding the transection plane and confirming viability before closing the abdomen. Bada-Bosch et al. described laparoscopic partial splenectomy assisted by ICG fluorescence in a pediatric case with a centralized splenic cyst, demonstrating that ICG defined both the transection plane and remnant perfusion. Extrapolation to trauma splenorrhaphy, where the decision between splenic salvage and splenectomy depends on confirming adequate residual perfusion, represents a clinically important application (18).

### 3.6 Current evidence and ongoing clinical trials

“Table 1 summarizes the principal ICG applications in acute settings and provides a pragmatic evidence-level classification based on the underlying study design, sample size, consistency of findings, and recommendations from the WSES consensus position paper. The evidence levels in Table 1 represent an author-developed pragmatic classification rather than a validated framework such as GRADE. Judgments considered study design, sample size, directness to acute-care populations, consistency of findings, and concordance with relevant consensus recommendations. Two authors independently reviewed each classification and resolved disagreements by discussion. Categories were downgraded when the evidence was indirect, heterogeneous, or limited to case reports or small observational series.”

**Table 1:** Summary of ICG applications in acute settings with evidence level.

Application	Setting	Evidence level*	Key source/recommendation
Biliary cholangiography (acute cholecystitis)	Emergency	High (meta-analyses, RCTs)	WSES 2025—Strong recommendation 1C (3)
Bowel perfusion/mesenteric ischemia	Emergency	Moderate (cohort, RCTs)	WSES 2025—Strong recommendation 1C; FLAMES trial (recruiting) (3, 70)
Anastomotic perfusion (colorectal)	Emergency/Elective	High (7 RCTs pooled)	SAGES 2025 meta-analysis: OR 0.58, *p* < 0.001 (4)
Strangulated hernia—bowel viability	Emergency	Low-moderate (case series)	WSES 2025—Strong recommendation 1C (3)
Penetrating abdominal trauma	Trauma	Low (case-control, trials)	Patel et al. 2024; NCT04534816 (completed) (6)
Burns depth assessment	Emergency/Trauma	Moderate (prospective RCT)	Wongkietkachorn et al.; WSES 2025 endorsed (3, 49)
Ureteric identification (emergency colonic resection)	Emergency	Moderate (cohort)	Soriano et al.; systematic review evidence (64, 65)
Extremity trauma/amputation level	Trauma	Low (case series)	Yang et al.; 30% viability cut-off (15)
TBI cortical perfusion (decompressive craniectomy)	Trauma	Very low (retrospective)	Kamp et al.; Forcione et al. (12, 13, 37)
Necrotizing fasciitis debridement	Emergency	Very low (case report, trials ongoing)	Galante ACS; NCT04839302; NCT06877793 (11, 61, 62)
Gonadal torsion viability	Emergency	Low (case series)	Nicholson et al.; Shirasaki et al. (17, 63)
Pancreatic necrosis debridement	Emergency	Very low (case reports)	Brito-Carmona et al. (36)
Thoracic duct/traumatic chylothorax	Trauma	Low (case series)	Vecchiato et al. (34)
War-related soft tissue/bowel injury	Trauma	Very low (retrospective case series)	Green et al. (19% management change) (16)
Hepatic trauma biliary leak (DCS)	Trauma	Very low (extrapolated)	Extrapolated from hepatobiliary elective data (31, 32)
Intestinal fistula detection (post-operative)	Post-operative	Very low (case report)	Peng et al. (20)
Partial splenectomy/splenic salvage	Trauma/Elective	Very low (case report)	Bada-Bosch et al. (18)

* Indicates direct acute-care evidence; entries without an asterisk are supported by indirect or elective evidence, early clinical evidence, or proposed use, as specified in the evidence classification.

However, the overall certainty of evidence remains limited for most indications, particularly in trauma and emergency surgery, where data are frequently derived from small cohorts, case series, feasibility studies, or extrapolation from elective practice. Evidence-based, indication-specific guidelines for many of the applications outlined in Table 1 are still lacking. Therefore, these classifications should be interpreted as a structured summary of the current evidence landscape rather than definitive recommendations. At present, the use of ICG in acute care surgery remains dependent on clinical context, equipment availability, local expertise, and the operating surgeon's judgment, while awaiting standardized protocols and higher-quality prospective data to guide implementation.

Table 2 contextualizes the current review within the evolving research landscape. By summarizing ongoing and recently completed registered trials, Table 2 highlights areas of active scientific interest, identifies applications moving from feasibility and observational evidence toward prospective evaluation, and directs readers to forthcoming data that may refine current practice. The results of these trials will be particularly important for determining whether the pragmatic uses of ICG outlined in this review can be translated into standardized protocols, indication-specific recommendations, and evidence-based guidelines for emergency and trauma surgery. Thus, Table 2 should be interpreted not only as a catalog of active studies, but also as a roadmap for the evidence expected to shape future implementation of ICG fluorescence-guided surgery in acute care settings (3, 4, 6, 70–75).

**Table 2:** Selected ongoing and recent clinical trials of ICG in acute settings.

Trial/Acronym	Focus	Status (2025)	NCT/Reference
FLAMES	Bowel ischemia (all-cause) mesenteric ischemia, strangulation	Recruiting	NCT06322862 (70)
Penetrating Abdominal Trauma ICG-FA	Penetrating bowel/intestinal injury	Completed	NCT04534816 (6)
NSTI ICG Kinetic Modelling	Necrotizing fasciitis diagnosis/debridement	Active (ongoing)	NCT04839302; NCT06877793 (61, 62)
ICG-COLORAL (Finland)	Colorectal anastomotic leak prevention	Published 2025	Rinne et al., JAMA Surg 2025 (5)
ICG Trauma Fractures/SSI	Bone/soft tissue perfusion in fractures	Completed	NCT04245111 (71)
Open Fracture Trauma ICG	Extremity fracture perfusion	Recruiting	NCT04416412 (72)
NIRST Compartment Syndrome	Compartment syndrome lower limb/forearm	Recruiting	NCT05672381 (73)
Abdominal Trauma ICG Feasibility	Penetrating abdominal trauma	Completed	NCT04534816 (6)
ICG Burns/Wound Healing (SPY)	Burn depth, amputation wound healing	Completed	NCT02766244 (74)
TBI Cerebral Perfusion (NIRS-ICG)	Subarachnoid/intracerebral hemorrhage, TBI	Recruiting	NCT01836848 (75)

Evidence was categorized by source context as: (1) direct acute-care evidence, derived from emergency, trauma, or other unplanned acute surgical populations; (2) indirect evidence, derived predominantly from elective populations but considered technically or clinically relevant to acute care; (3) early clinical evidence, limited to small cohorts, feasibility studies, or case reports; or (4) proposed use, based on physiological rationale or extrapolation without substantive acute-care clinical evidence. These labels are reported in Table 1 and used throughout the narrative synthesis. To prevent applications with markedly different evidentiary maturity from being interpreted as equivalent, indications were additionally categorized as practice-ready, emerging, investigational, or proposed. Practice-ready applications have direct acute-care evidence and/or consensus support sufficient for selected clinical use; emerging applications have promising but limited clinical evidence; investigational applications are undergoing prospective evaluation or remain supported mainly by feasibility data; and proposed applications are based largely on indirect evidence or physiological rationale. This classification is intended to guide interpretation, table organization, figure design, and research prioritization rather than prescribe an universal clinical practice. To make the evidence-maturity distinction explicit, we added a four-tier categorization of ICG applications in acute settings (Table 3 and Figure 6).

**Table 3:** Evidence maturity category to clearly describe current applications into 4 categories.

Evidence-maturity category	Definition	Included applications
Established/practice-ready	Applications with direct acute-care evidence and/or consensus support sufficient for selected clinical use.	Fluorescent cholangiography in acute cholecystitis; bowel perfusion assessment in acute mesenteric ischemia, strangulation, incarcerated hernia, and volvulus; perfusion-guided emergency bowel resection and selected colorectal anastomosis.
Emerging	Applications with promising but still limited clinical evidence, usually from cohort studies, feasibility studies, case-control data, or systematic reviews with heterogeneous evidence.	Ureteric identification during emergency pelvic or colorectal surgery; burn-depth assessment; lower-extremity trauma, amputation level, debridement, flap or stump viability; penetrating abdominal trauma; gonadal torsion viability; pancreatic necrosis debridement; thoracic duct mapping for chylothorax.
Investigational	Applications undergoing active prospective evaluation or supported mainly by early feasibility data, pilot studies, or technology-development work.	Necrotizing soft-tissue infection diagnosis and debridement guidance; traumatic brain injury and neurocritical-care cerebral perfusion monitoring; compartment syndrome or bedside limb-perfusion monitoring; AI-assisted quantitative fluorescence interpretation.
Proposed	Applications based mainly on physiological rationale, indirect elective evidence, or extrapolation, with insufficient direct acute-care validation.	Hepatic trauma biliary-leak detection after damage-control surgery; splenic salvage or partial splenectomy in trauma; appendiceal base or stump perfusion assessment; postoperative fistula confirmation; stoma viability assessment; future targeted fluorophores and multimodal fluorescence-fusion approaches.

**Figure 6:**
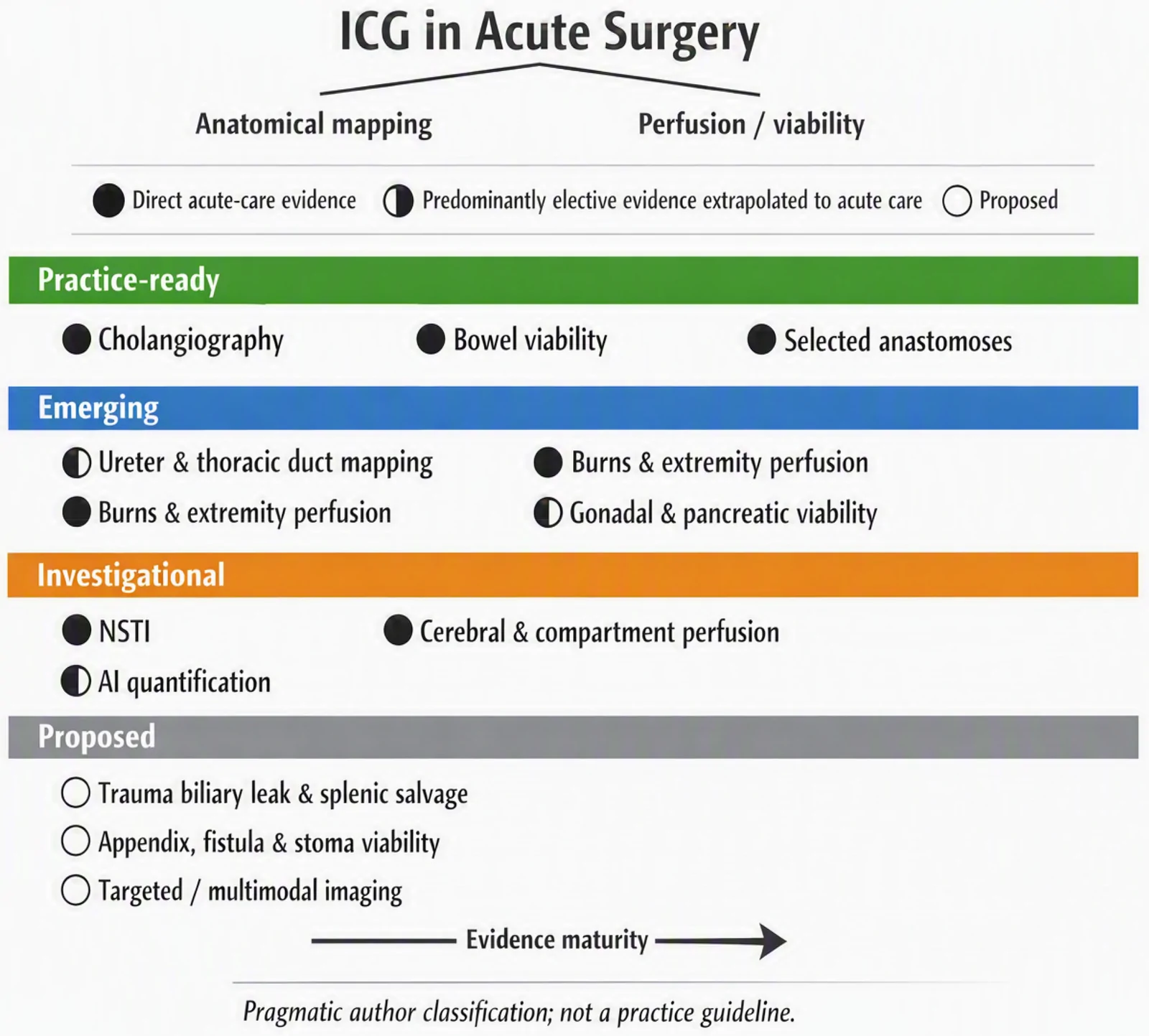
ICG in acute surgery: evidence spectrum. Evidence maturity increases from proposed to investigational, emerging, and practice-ready applications. This author-developed classification supports interpretation and research prioritization and is not a practice guideline.

### 3.7 Practical implementation guide

#### 3.7.1 Setup and equipment

Implementation of ICG-guided surgery requires access to a NIR-capable laparoscopic, open-field, or handheld imaging platform and readily available ICG for reconstitution. Handheld NIR devices extend use beyond laparoscopy to open trauma laparotomies, wound debridement, and bedside monitoring, which is particularly relevant when minimally invasive access is not feasible. After equipment acquisition, the incremental reagent cost per case is low, typically less than USD 5 per test. However, adoption should be guided by formal cost analysis that weighs added equipment, maintenance, consumables, training, and workflow costs against potential benefits, including shorter operative time, fewer conversions, reduced leak- or ischemia-related reoperations, avoidance of unnecessary resections or stomas, improved wound or stump decision-making, and greater workflow efficiency. Available consensus evidence suggests that downstream clinical and operational savings may offset upfront costs, although local case volume, device availability, institutional workflow, and training capacity should inform implementation decisions (3, 4, 16, 23, 24). Table 4 summarizes the ICG Dosing and Administration Summary by Acute Application.

**Table 4:** ICG dosing and administration summary by acute application.

Application	Route	Dose	Timing	Notes
Biliary cholangiography (acute cholecystitis) standard approach	IV	0.1 mg/kg IV	30–60 min pre-op	Routine fluorescent cholangiography; hepatic background may limit signal (3, 27, 29)
Biliary cholangiography (acute cholecystitis) difficult/inflamed cases	IB	5 mL of 2.5 mg/mL	Intra-operative	Use when severe inflammation or hepatic background limits IV visualization; requires cystic duct patency (3, 27, 29)
Bowel perfusion/anastomosis	IV	2.5–7.5 mg; commonly 5 mL of 2.5 mg/mL	2–3 min before assessment	Repeat after anastomosis if needed; maximum total dose 0.25 mg/kg (3, 9, 10, 35)
Ureteric identification	IU (retrograde)	2 mL of 2.5 mg/mL per ureter	Start of procedure	Requires cystoscopy with or without stent; signal may last up to 8 h (64, 65)
Burns depth assessment	IV	0.1–0.5 mg/kg	Intra-operative	Use video-angiography mode; assess perfusion kinetics, not only peak signal (46, 49, 50)
Amputation level/flap viability	IV	5–10 mg; approximately 0.1–0.2 mg/kg	Intra-operative	Relative perfusion value >30% supports viability (15, 40–45)
Thoracic duct identification	SC inguinal	0.5 mg/kg in 1–5 mL	30–60 min before thoracoscopy	Prone positioning may optimize lymphatic uptake (34)
Gonadal torsion viability	IV	2–5 mg; 2.5 mg/mL	After detorsion	Visualization in approximately 60 s; supports preserve-versus-resect decisions (17, 63)
Pancreatic necrosis debridement	IV	3 mL IV	Intra-operative	Used to confirm viable residual pancreatic tissue (36)
Fistula detection (post-operative)	Oral/IV	25 mg in 50 mL orally; 5 mg IV	Bedside, post-operative	NIR camera detects ICG in drain fluid; no sedation required (20)

#### 3.7.2 Dosing summary by application

#### 3.7.3 Workflow integration in the emergency setting

Successful ICG integration in emergency surgery requires preparation and a team-based protocol to ensure optimal outcome. The NIR camera or laparoscope should be confirmed to be NIR-capable before the case begins, and ICG should be reconstituted and held in readiness for IV or IB injection. In trauma cases, early communication between the scrub nurse and anesthetist ensures that ICG can be administered at the critical decision point without interrupting the resuscitation (3, 4, 24).

The WSES consensus recommends laparoscope positioning at <5 cm from the target and perpendicular (90-degree) orientation to the target tissue to maximize signal quality. In open surgery, the equivalent principle applies to handheld device positioning. Surgeons should be aware that tissue penetration is limited to 5–10 mm; structures beneath this depth require anatomical dissection before ICG can reliably identify them. Obesity, significant tissue edema, and active bleeding reduce ICG signal quality and should prompt consideration of adjusted dosing or delayed reassessment (3, 25).

#### 3.7.4 Summary algorithm for ICG use in the emergency setting

In acute care surgery, ICG fluorescence is best understood through two practical domains: anatomical mapping and perfusion assessment. Anatomical mapping supports identification of vulnerable structures when inflammation, bleeding, distorted planes, or contamination limit white-light assessment, particularly in fluorescent cholangiography, ureteric identification, thoracic duct mapping, and selected biliary-leak evaluation. Perfusion assessment has broader clinical relevance and includes bowel viability in mesenteric ischemia, strangulation, volvulus, and incarcerated hernia; assessment before and after emergency anastomosis; pancreatic debridement guide, staged reassessment in damage control surgery; and selected open-field applications such as burns, necrotizing soft tissue infection, flap or stump viability, gonadal torsion, pancreatic necrosis, and splenic preservation (3, 4, 6, 9, 10, 17, 25–36, 49, 50, 59–65, 67–70).

Supplementary Figure 1 shows a step-wise summary of intra-operative ICG use in emergency and trauma surgery**.** After confirming safety, equipment availability, and a clinically meaningful indication, the surgeon determines whether the primary problem is anatomical identification or tissue viability. The route of administration is then matched to the indication: intravenous delivery for most perfusion applications, with intra-biliary, intra-ureteric, subcutaneous inguinal, or oral administration reserved for specific scenarios. Across all uses, fluorescence findings should complement, not replace, operative judgment. The most practice-ready applications are fluorescent cholangiography in acute cholecystitis, bowel perfusion assessment in mesenteric ischemia or strangulation, and perfusion-guided emergency bowel anastomosis (3, 4, 9, 10, 25–30, 64, 65, 67–70).

Overall, current evidence supports ICG fluorescence as a practical adjunct for image-guided decision-making in emergency and trauma surgery. Its strongest near-term role is in improving anatomical orientation and refining perfusion-based decisions when conventional visual and tactile cues are limited. Although the most mature applications include fluorescent cholangiography, bowel viability assessment, and perfusion-guided emergency anastomosis, emerging uses in penetrating trauma, necrotizing soft tissue infection, damage control surgery, organ salvage, and neurocritical care suggest a broader movement toward precision acute care surgery. Future adoption will depend on standardized protocols including proper training, objective fluorescence interpretation, and prospective validation across emergency surgical populations (1–4, 6, 9, 10, 35, 59–63, 67–70).

### 3.8 ICG limitations, standardization, and current challenges

Against this background of expanding clinical applicability, it is equally important to define the methodological, technical, and implementation-related constraints that currently limit the broader, standardized adoption of acute care surgery (3, 7, 19, 24, 66).

#### 3.8.1 Subjectivity of qualitative intra-operative signal interpretation

The most significant limitation of current ICG-guided surgery is the subjectivity in interpreting the fluorescence signal. The standard workflow involves the surgeon visually assessing the fluorescence image and making a binary (resect/preserve) or graded (adequate/marginal/inadequate) judgment based on the perceived signal intensity and distribution. This is susceptible to inter-observer variability, device sensitivity differences, and confounders such as tissue thickness, inflammation, and background signals. For bowel perfusion assessment, this means that the same fluorescence pattern may be interpreted differently by different surgeons or by the same surgeon on different occasions (3, 19, 66).

Further studies are needed to correlate ICG imaging patterns with histological evidence of ischemia. Operators must understand that ICG fluorescence provides additional information (not a replacement for) clinical judgment and that both under- and over-reliance carry risk (3).

#### 3.8.2 Lack of standardized protocols in acute settings

The dosing, timing, and interpretation of ICG signals have not been standardized in emergency contexts. This heterogeneity, reflecting the diversity of applications, post-operative operations, imaging devices, and clinical endpoints, makes comparisons across studies difficult and prevents definitive evidence-based dosing recommendations. The recent consensus paper represents the first attempt at standardization through a structured Delphi process but acknowledges that most of the recommendations are based on low-quality evidence (3).

#### 3.8.3 Limitations in acute contexts

Several factors specific to emergency settings reduce ICG performance and the decision to utilize it when considered critically. Hemodynamic instability and vasopressor use alter tissue perfusion and ICG distribution, potentially generating false-positive or false-negative results. Hepatic dysfunction, common in patients with sepsis or polytrauma, prolongs ICG clearance and increases background fluorescence. Massive blood transfusion, coagulopathy, and hemodilution affect ICG plasma protein-binding. These confounders are rarely present in elective surgical populations from which most ICG dosing guidelines are derived (3, 25).

#### 3.8.4 Equipment availability and training

The ARIES-WSES international survey on knowledge, attitudes, and practices (KAP) of ICG fluorescence in emergency surgery (De Simone et al., Updates Surg, 2024) identified equipment availability and the absence of training as the two leading barriers to adoption (7). Unlike elective settings where NIR-capable laparoscopes are now standard in most high-volume centers, emergency operating rooms, especially in resource-limited environments, may not have this equipment readily available. Education and simulation-based training for emergency surgeons on the ICG technique and signal interpretation are needed (3, 7).

### 3.9 Future directions

If these current barriers define the limits of present-day implementation, they also clarify where the field must advance to achieve broader, more objective, and more scalable integration into intra-operative care (3, 19, 66, 76–83).

#### 3.9.1 Quantitative fluorescence and artificial intelligence

The next frontier of ICG-guided surgery is the objectification of fluorescence signal interpretation through quantitative analyses. Instead of relying on a surgeon's visual judgment, the software extracts time-fluorescence curves from the NIR video stream and derives kinetic parameters—peak fluorescence, time-to-peak, wash-in and wash-out slopes, and area under the curve—that reflect microvascular perfusion at the pixel level. Multiple commercial and research-grade quantitative ICG software systems have been developed to analyze ICG images (19, 66).

The integration of artificial intelligence (AI) and machine learning with ICG quantification is an emerging field that is rapidly advancing. Malik et al. demonstrated an AI-enhanced perfusion assessment based on ICG kinetics for anastomotic leak prediction in left-sided colorectal surgery (19). AI models trained on fluorescence video data have been shown to outperform visual surgeon assessments in identifying suboptimal perfusion patterns associated with anastomotic leakage. Diana et al.'s fluorescence-based enhanced-reality approach overlays quantitative perfusion maps on white-light images in real time, enabling simultaneous anatomical and perfusion guidance (66). The future of ICG in acute surgery will likely involve integrated AI-assisted decision support tools that provide surgeons with objective, real-time perfusion scores rather than raw fluorescence images, directly addressing limitations of subjectivity (19, 66).

#### 3.9.2 New fluorophores and targeted agents

While ICG remains the primary FDA-approved fluorophore for surgical guidance, next-generation agents are under active development. Nerve-specific fluorophores, including optimized oxazine-based probes, are being developed for intra-operative nerve visualization and nerve-sparing surgery. Renally excreted fluorophores, including ZW800-1 and UreterGlow-11, may permit non-invasive ureteric visualization without retrograde catheterization. Tumor-specific targeted fluorophores based on monoclonal antibody conjugates are being evaluated in oncological surgery. In the acute context, infection-specific fluorophores with preferential uptake in bacterially colonized tissue could transform the diagnosis and treatment of necrotizing soft tissue infections—making fluorescence guidance more specific without relying only on perfusion absence as a surrogate marker (76–80).

#### 3.9.3 Hyperspectral imaging and multimodal fusion

Hyperspectral imaging (HSI) acquires tissue spectra across multiple wavelength bands without exogenous fluorophores, providing perfusion and oxygenation data through tissue optical properties. Combined ICG-FA and HSI may provide complementary perfusion information, and emerging surgical studies suggest that multimodal approaches can support resection guidance and tissue-perfusion quantification. The integration of ICG-FA with other real-time surgical navigation tools, such as ultrasound, augmented reality overlays, extended reality platforms, and fluorescence-guided robotic systems, represents a broader direction of intra-operative precision surgery, applicable to trauma and emergency contexts as much as to elective oncological procedures (81–83).

Future priorities include standardized protocols, prospective multicenter validation, objective fluorescence interpretation, and AI-supported quantitative perfusion analysis. These steps are necessary to translate the pragmatic applications outlined in this review into reproducible workflows, indication-specific recommendations, and evidence-based guidelines for precision emergency and trauma surgery (3, 4, 19, 24, 66, 70–83). Figure 7 summarizes our proposed decision-making algorithm for the utility of ICG in acute surgical settings.

**Figure 7:**
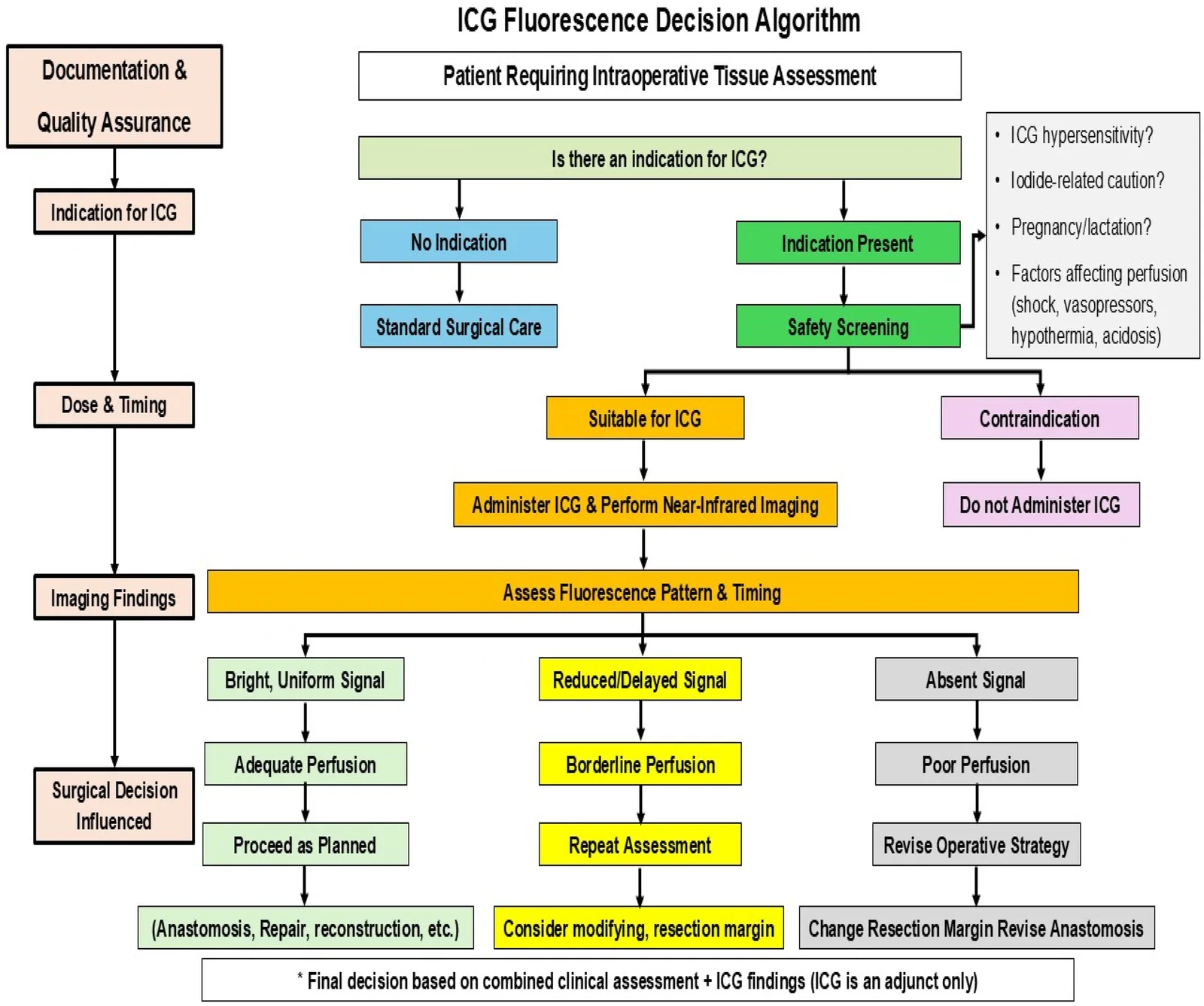
A summary of the proposed decision making algorithm for the utility of ICG in acute surgical settings.

## 4 Limitations

This review has methodological limitations. Publication and selective-reporting bias may favor successful or management-changing uses of ICG. The included literature is heterogeneous in populations, indications, imaging platforms, dosing, timing, fluorescence interpretation, outcome definitions, and study design. Evidence for several emerging applications is derived from small observational cohorts, feasibility studies, or case reports, and some acute-care uses are extrapolated from elective populations. The search relied on one principal bibliographic database, supplemented by trial registries, guidelines, and citation searching, and may have missed non-indexed or non-English reports. Finally, no formal risk-of-bias instrument or certainty-of-evidence framework was applied; therefore, the evidence categories should be interpreted as pragmatic descriptors rather than definitive recommendations.

## 5 Conclusions

ICG fluorescence-guided surgery is a safe, relatively low-cost, and rapidly deployable adjunct for emergency and trauma surgery. Its main value is the provision of real-time anatomical and perfusion information when conventional visual and tactile assessment is limited by inflammation, ischemia, contamination, bleeding, or distorted operative planes. The most mature acute-care applications are fluorescent cholangiography in acute cholecystitis, bowel viability assessment in mesenteric ischemia or strangulation, and perfusion-guided emergency bowel resection and anastomosis.

Broader reported uses, including burns, extremity and flap viability, necrotizing soft tissue infection, damage control surgery, penetrating trauma, organ salvage, fistula detection, and selected neurocritical-care applications, support pragmatic integration when ICG addresses a defined intra-operative uncertainty. However, evidence for many indications remains heterogeneous and often derives from small cohorts, case series, feasibility studies, or extrapolation from elective practice. Therefore, ICG should complement rather than replace operative judgment, with use guided by indication, local expertise, workflow readiness, and cost-benefit considerations and research priority should consider prospective multicenter as well as local registry in the institutes that uses ICG FGS to define its role in practice acute settings.
