# Supplementary material for: Indocyanine green fluorescence-guided surgery in acute settings: state-of-the art
Source: Front Surg. 2026 Sep 28;13:1962138. doi: 10.3389/fsurg.2026.1962138 (PMC13655528; doi:10.3389/fsurg.2026.1962138)
Supplement: Supplementary file 1 [file Supplementaryfile1.docx]

**Suppl Figure 1. Journal-style summary algorithm for intraoperative ICG use in emergency and trauma surgery**

**ENTRY QUESTION**

Can ICG be used safely and meaningfully in this case? Avoid known ICG hypersensitivity.[3,25]

Confirm NIR-capable equipment, ICG availability, and the intended route.[3,4,24]

The interpretation of these results should be cautious in patients with hepatic dysfunction, severe inflammation, obesity, shock, or vasopressor-dependent states.[3,25]

**DECISION NODE**

**Anatomical mapping** versus **Perfusion assessment**

| **ANATOMICAL MAPPING**  **Fluorescent cholangiography in** acute cholecystitis and difficult gallbladder.[3,25-30]  **Ureteric identification:** emergency colorectal or pelvic dissection.[33,64,65]  **Thoracic duct mapping:** chylothorax or thoracic trauma.[34]  **Selected biliary leak mapping:** hepatic trauma re-examination or unclear biliary anatomy.[3,31,32] | **PERFUSION ASSESSMENT**  **Bowel viability:** AMI, strangulation, volvulus, and incarcerated hernias.[3,9,10,67-70]  **Anastomotic perfusion:** before transection and after reconstruction.[4,5,8,35]  **Trauma/damage control:** penetrating bowel injury and second-look laparotomy.[3,6]  **Soft tissue/burns/limb salvage:** NSTI, burn depth, and flap or stump viability.[15,16,40-62,71-74]  **Selective organ salvage in** gonadal torsion, pancreatic necrosis, and splenic salvage.[17,18,36,63] |
| --- | --- |

**TECHNICAL MATCHING**

**IV** for most perfusion applications.[3,9,10,35]

**Intrabiliary** for difficult acute cholecystitis when the hepatic background limits contrast.[3,27,29]

**Intraureteric** for ureter mapping.[64,65]

**Subcutaneous inguinal** for thoracic duct visualization.[34]

**Oral** assessment of selected postoperative fistulas.[20]

**INTERPRETATION**

Fluorescence should refine, rather than replace, clinical judgment.[3,19,66]

The signal should be integrated with tissue appearance, hemodynamics, bleeding, contamination, vasopressor exposure, and overall operative strategy.[3,25]

**HIGHEST-YIELD CURRENT USES**

**Most practice-ready:** fluorescent cholangiography in acute cholecystitis, bowel viability assessment in mesenteric ischemia or strangulation, and perfusion-guided emergency bowel anastomosis.[3,4,9,10,25-30,35,67-70]

**Promising but evolving:** NSTI, penetrating abdominal trauma, TBI monitoring, pancreatic necrosis, and organ salvage.[6,11-13,17,18,36,59-63,75]
